# Role of tumor-derived exosomes in metastasis, drug resistance and diagnosis of clear cell renal cell carcinoma

**DOI:** 10.3389/fonc.2022.1066288

**Published:** 2022-12-21

**Authors:** Tiancheng Jiang, Zepeng Zhu, Jiawei Zhang, Ming Chen, Shuqiu Chen

**Affiliations:** ^1^ Department of Urology, Zhongda Hospital, Southeast University, Nanjing, China; ^2^ Department of Medical College, Southeast University, Nanjing, China

**Keywords:** clear cell renal cell carcinoma, tumor-derived exosomes, metastasis, diagnosis, drug resistance

## Abstract

Renal cancer is one of the most extensively studied human tumors today, with clear cell renal cell carcinoma accounting for approximately 80% of all cases. Despite recent advances in research on clear cell renal cell carcinoma, advanced distant metastasis of the disease, delay in diagnosis, as well as drug resistance remain major problems. In recent years, as an important mediator of material and information exchange between cells in the tumor microenvironment, exosomes have attracted widespread attention for their role in tumor development. It has been reported that tumor-derived exosomes may act as regulators and have an important effect on the metastasis, drug resistance formation, and providing targets for early diagnosis of clear cell renal cell carcinoma. Therefore, the extensive study of tumour-derived exosomes will provide a meaningful reference for the development of the diagnostic and therapeutic field of clear cell renal cell carcinoma. This article reviews the biological role and research progress of tumor-derived exosomes in different aspects of premetastatic niche formation, tumor angiogenesis, and epithelial-mesenchymal transition during the progression of clear cell renal cell carcinoma. In addition, the role of tumor-derived exosomes in the development of drug resistance in clear cell renal cell carcinoma is also addressed in this review. Furthermore, recent studies have found that cargoes of exosomes in serum and urine, for example, a series of miRNAs, have the potential to be biological markers of clear cell renal cell carcinoma and provide meaningful targets for early diagnosis and monitoring of tumors, which is also covered in this article.

## 1 Introduction

Kidney cancer is the third most common cancer of the urinary system. According to the World Health Organization, renal cancer ranked 16th among all cancers worldwide in terms of new cases and deaths in 2020 (1). Clear cell renal cell carcinoma (ccRCC), a renal cortical tumor characterized by malignant epithelial cells, is the most common type of kidney cancer, accounting for 80% of total cases, and other subtypes are mainly papillary renal cell carcinoma, suspicious cell carcinoma, clear cell papillary renal cell carcinoma, and MIT family translocation-associated renal cell carcinoma (2). At present, the global morbidity and mortality of ccRCC continue to rise, and although recent progress has been made in the study of ccRCC, its prognosis is still poor. The diagnosis and treatment of ccRCC also involves many social factors, such as racial imbalances, like the racial disparities that exist among patients undergoing robotic radical nephrectomy (3). In fact, more than 60% of ccRCCs are found incidentally. Despite improvements in imaging techniques, more than 30% of patients have already been found to have tumor metastases, especially bone metastases, lung metastases, accounting for approximately 15% of total cases at the time of diagnosis (4–6). At the same time, the drug resistance of ccRCC is also one of the main reasons for their clinical treatment failure, especially metastatic ccRCC. For example, vascular endothelial growth factor (VEGF), mammalian target of rapamycin (mTOR) inhibitors, and RAF kinase have been used to treat ccRCC (7), but these drugs often encounter the problem of drug resistance in clinical application. Therefore, if ccRCC can be diagnosed and monitored early, it is beneficial to improve the clinical management of ccRCC, perform medical or surgical intervention before tumor cell metastasis, and prolong the overall survival of ccRCC patients. There are no specific molecular markers for ccRCC for clinical use (8). However, it has been found that tumor-released extracellular vesicles, for example, exosomes, may represent a new class of biomarkers for liquid biopsy, providing a meaningful target for the early diagnosis and monitoring of ccRCC (9).

**Figure 1:**
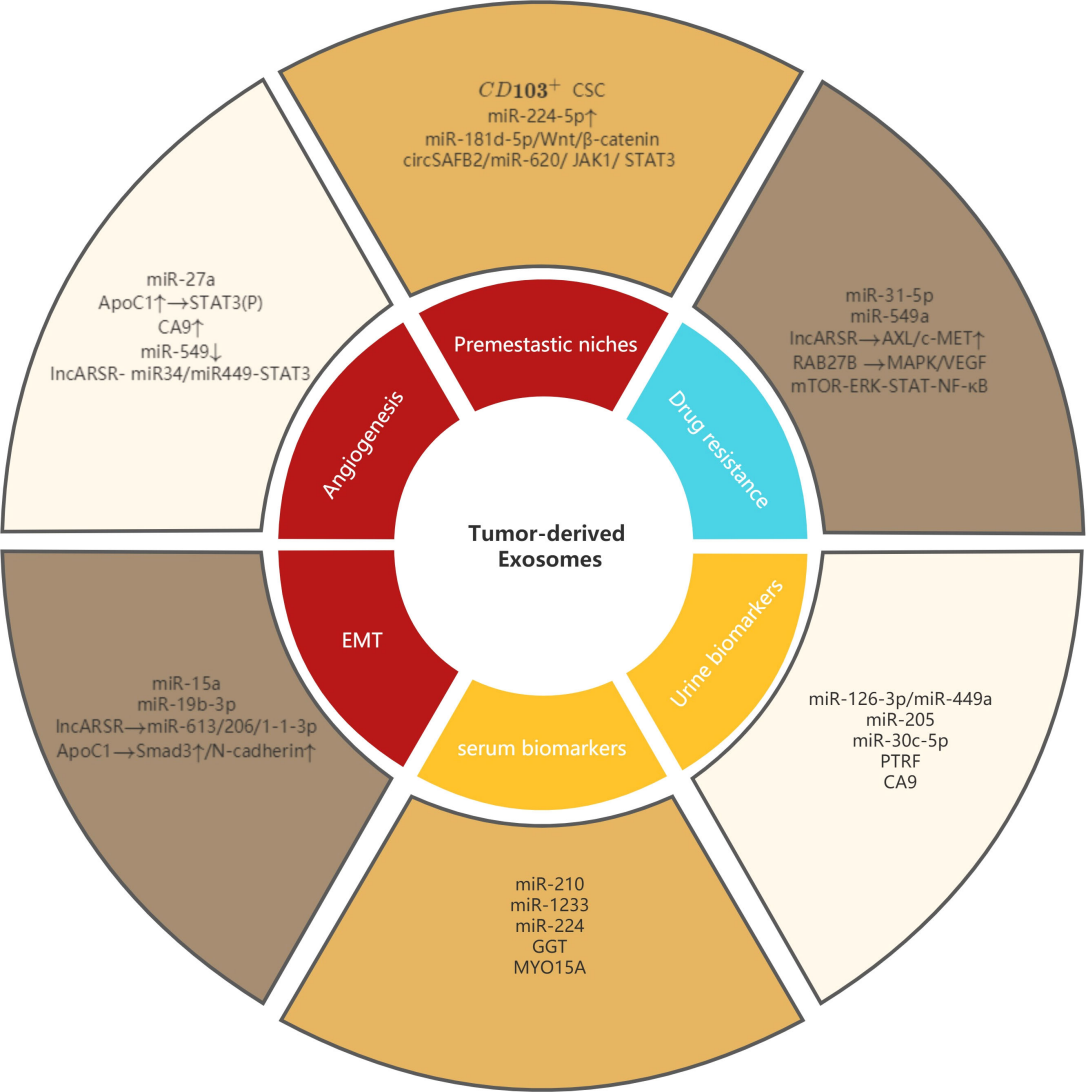
Role of tumor-derived exosomes in metastasis, diagnosis and drug resistance of ccRCC. Tumor-derived exosomes involved in premetastatic niches, angiogenesis, EMT and drug resistance of ccRCC. Tumor-derived exosomes can also be used as serum biomarkers and urine biomarkers in ccRCC.

Extracellular vesicles (EVs) are a collective term for various vesicular structures with membrane structures released by cells. According to their different diameters, they can be divided into three types: exosomes, microvesicles, and apoptotic bodies (10). Among them, exosomes are goblet extracellular vesicles with a diameter of 40-100 nm, surrounded by a lipid bilayer membrane, which is currently a research hotspot. Exosomes were found in nearly all body fluids, including serum, urine, cerebrospinal fluid, saliva, and tears. Human normal cells as well as tumor cells can secrete exosomes, which play an important role in intercellular communication, and the cargo transported by them includes proteins, mRNAs, miRNAs and signaling molecules. Exosomes regulate the physiological state of cells by carrying and transmitting signaling molecules and are involved in antigen presentation, cell differentiation, growth, tumor immune response, and migration and invasion of tumor cells (11). Many researches have shown that tumor-derived exosomes play an important role in the occurrence, progression and metastasis, immune escape, drug resistance and early diagnosis of ccRCC by transporting different cargoes and mediating related signaling pathways (12–16), and these findings provide us with meaningful targets for further study of ccRCC (shown in **Figure 1**).

## 2 Tumor-derived exosomes promote metastasis of ccRCC

### 2.1 Involved in the formation of premetastatic niches

Paget first proposed the’seed and soil’ theory in 1889, which likens metastatic tumor cells as seeds and potential metastatic sites as soil to explain that tumor metastasis has organ tropism (17). There is increasing evidence that primary tumors can remotely regulate the formation of the tumor microenvironment in secondary organs by secreting a variety of cytokines, allowing tumor cells to colonize the circulation. This local tumor microenvironment is known as the premetastatic niche (PMN) (18). The establishment of PMN is closely related to the release of tumor-secreted factor (TSF) and tumor-secreted exosome (TSE) from the primary tumor, and TSE is considered to be the main driver of PMN formation. A key step in the process of promoting the dissemination of tumor cells from the primary site to the metastatic site is the enhancement of vascular permeability. A study by Zeng showed that exosomes secreted by colorectal cancer (CRC) cells are rich in miR-25-3p, which can disrupt the tight junctions of vascular endothelial cells by targeting the transcription factors KLF2 and KLF4, leading to tumor angiogenesis increased permeability, thereby promoting tumor metastasis (19).

In ccRCC, the ability to promote PMN formation mainly comes from EVs secreted by cancer stem cells (CSC). Renal CSC EVs contain a variety of miRNAs, which may be involved in biological pathways related to cell growth and cell matrix adhesion (20). Studies have shown that CSC exosomes from metastatic ccRCC patients accelerate ccRCC cell proliferation and lung metastasis. Especially CSC exosomes expressing integrin CD103. Wang et al. found that the proportion of exosomes in the blood of metastatic ccRCC patients to total exosomes was significantly higher than that of non-metastatic ccRCC patients. CSC exosomes are more organotropic than CSC exosomes and play an important role in targeting distant organs and forming PMN (13).

Furthermore, in the PMN of ccRCC, besides tumor cells, there are a large number of stromal cells and immune cells. Among them, cancer-associated fibroblasts (CAFS) are the main stromal components in the ccRCC tumor microenvironment and are closely related to tumor progression (21). Liu et al. found that miR-224-5p was enriched in CAFS-derived exosomes in PMN, and miR-224-5p could be transferred from CAFS-derived exosomes into ccRCC cells, and then further secreted in the form of exosomes. After upregulation of miR-224-5p, the number of ccRCC cells undergoing migration and invasion was significantly increased (22). Fu also found that in the process of ccRCC metastasis, CAFS-derived exosomes can enter ccRCC cells through internalization, inhibit tumor cell apoptosis and regulate cell cycle, and promote the progression of ccRCC (12). A new study found that CAFS-derived exosomes directly inhibit ring finger protein 43 (RNF43) expression and activate the Wnt/β-catenin signaling pathway by delivering miR-181d-5p, thereby promoting RCC stemness and progression (23). Additionally, Huang et al. found that circSAFB2 delivered by ccRCC-derived exosomes mediates the polarization of M2 macrophages *via* the miR-620/JAK1/STAT3 axis, thereby remodeling the tumor microenvironment and ultimately promoting ccRCC metastasis (24).

In conclusion, tumor-associated exosomes are involved in the formation of PMN, providing suitable environmental conditions for the metastasis of ccRCC.

### 2.2 Involved in angiogenesis of ccRCC

It is well known that tumor angiogenesis is an important step in the metastatic progression of malignant tumors. Relevant studies have shown that tumor-derived exosomes play an important role in tumor angiogenesis. For example, Yang et al. demonstrated that exosomal miR-130a promoted gastric cancer angiogenesis by targeting C-MYB in vascular endothelial cells, thereby promoting tumor metastasis (25). Huang et al. found that exosomes derived from liver cancer cells participate in the angiogenesis of liver cancer by carrying circRNA-100,338, and then promote the lung metastasis of liver cancer (26). In addition, exosomal miR-27a from pancreatic cancer cells can participate in the angiogenesis of human microvascular endothelial cells by targeting BTG2 (27).

In ccRCC, tumor-derived exosomes can also participate in tumor angiogenesis by delivering a variety of miRNA and protein molecules. A recent study found that gene secreted frizzled-related protein 1 (SFRP1) was underexpressed and miR-27a was overexpressed in ccRCC cells. Grange et al. have proved that miR-27a acts as an oncomiR. CcRCC cell-derived exosomes accelerate tumor growth and angiogenesis *in vivo* by delivering miR-27a, downregulating the expression of the tumor suppressor gene SFRP1, while increasing the expression levels of vascular endothelial growth factor and tumor necrosis factor-alpha (TNF-α) (28). According to the Oncomine database, we found that among all cancer types, the greatest upregulation of apolipoprotein C1 (ApoC1) was observed in kidney cancer samples. Li et al. demonstrated that ccRCC-derived exosomes can mediate the transfer of ApoC1 from ccRCC cells to tumor vascular endothelial cells, which in turn promotes angiogenesis by activating the transcription factor STAT3, and promotes the metastasis of ccRCC cells (29). In addition, studies have also shown that carbonic anhydrase 9 (CA9) in exosomes released from hypoxic ccRCC cells can enhance angiogenesis in the tumor microenvironment, thereby promoting cancer progression (30). Interestingly, miRNAs delivered by tumor-derived exosomes may also play a role in inhibiting ccRCC angiogenesis. Xuan et al. found that miR-549a expression was lower in tyrosine kinase inhibitor (TKI)-resistant ccRCC cells and their derived exosomes. They demonstrated that exosomal miR-549a could reduce the expression level of hypoxia-inducible factor 1 alpha subunit (HIF1α) by binding to the 3’-UTR region of HIF1α mRNA, which in turn attenuate tumor angiogenesis and endothelial cell migration in ccRCC (31). A new study found that ccRCC-derived exosomes can activate the miR34/miR449-STAT3 signaling pathway by delivering lncARSR, which in turn promotes the transformation of M1 macrophages to M2 and enhances the phagocytic ability of macrophages, which in turn induces angiogenesis and ultimately promotes the development and progression of tumors (32). In summary, ccRCC cell-derived exosomes participate in tumor angiogenesis by delivering a variety of miRNA and protein molecules, thereby affecting the metastasis process of ccRCC.

### 2.3 Involved in EMT of ccRCC

Epithelial-mesenchymal transition (EMT) is the process by which epithelial cells (E cells) lose their polarity and transform into mesenchymal cells (M cells). EMT is widely recognized as a key event in tumor invasion and distant metastasis. In part, this process occurs through the degradation of cell adhesion junctions and changes in gene expression, resulting in an enhanced ability of tumor cells to invade tissues and metastasize (33). At present, a large number of studies have shown that tumor-derived exosomes can mediate EMT of tumor cells by delivering a variety of miRNA and protein molecules, and promote the ability of tumor cells to invade and migrate. For example, He et al. found that exosomes derived from non-small cell lung cancer cells, targeting the mTOR pathway by delivering miR-499a-5p, could promote the EMT process of lung adenocarcinoma (34). You et al. also demonstrated that exosomes derived from cervical cancer cells, stimulated by TGF-β1, can deliver miR-663b targeting MGAT3 to promote EMT and metastasis of cervical cancer (35).

In ccRCC, the relationship between tumor-derived exosomes and EMT has also received attention. Li et al. demonstrated that miR-15a was upregulated in exosomes derived from ccRCC cells. Furthermore, exosomal miR-15a can enhance EMT activity in ccRCC by downregulating BTG2 gene and promoting PI3K/AKT signaling pathway activity (36). Wang et al. demonstrated that in metastatic ccRCC patients, CSC exosomes induced EMT by transporting miR-19b-3p to cancer cells and inhibiting the expression of PTEN gene (13). Hu et al. found that lncHILAR was abundant in the cytoplasm of ccRCC cells, and exosomes were its main carrier. At the same time, they found that exosomal lncHILAR was associated with EMT gene bank markers through gene enrichment analysis. Under normoxia and hypoxia, knockdown of lncHILAR could reverse EMT, indicating that exosomal lncHILAR could promote ccRCC metastasis by inducing EMT (37). Li et al. also found that the overexpression of exosomal ApoC1 increased the mRNA levels of Smad3 and N-cadherin on ccRCC cells, which in turn promoted the EMT process and increased the migration and invasion abilities of ccRCC (29). In conclusion, ccRCC cell-derived exosomes can promote the EMT process of ccRCC by delivering various cargos, especially miRNA, and then promote the metastasis of ccRCC.

## 3 Tumor-derived exosomes promote drug resistance in ccRCC

As mentioned above, tumor-derived exosomes do play a non-negligible role in the metastasis of ccRCC. Moreover, tumor-derived exosomes can further cause drug resistance and immune escape of tumor cells on this basis. The development of drug resistance is one of the main causes of clinical treatment failure in tumors. At present, renal cancer is not sensitive to radiotherapy and chemotherapy, and patients with advanced renal cancer treated with targeted agents have a low rate of complete remission. In most patients with advanced or metastatic ccRCC, systemic first-line therapy mainly includes immune checkpoint inhibitors, TKIs, and mTOR inhibitors (38). Among them, TKI is one of the first-line therapies for advanced ccRCC, and most patients will eventually develop TKI-resistant ccRCC and then develop metastasis after 6-15 months (39). Numerous studies have shown that drug-resistant tumor cells secrete exosomes containing genetic information of multiple drug-resistance-related proteins, which in turn leads to the acquisition of drug resistance by other drug-sensitive tumor cells (40). In addition, tumor-specific exosomes have also been found to play an important role in immunosuppression (41, 42). For example, exosomes secreted by kidney cancer cells can induce immune responses in T cells to trigger apoptosis of activated T lymphocytes by activating the caspase pathway (16). In ccRCC patients, tumor-derived exosomes play an important role in the formation of drug resistance in ccRCC, and exosomes can mediate drug resistance by delivering miRNA, lncRNA, and protein molecules. The role of tumor-derived exosomes in drug resistance in ccRCC is specifically addressed in the following section.

### 3.1 Exosomal miRNAs mediate ccRCC drug resistance

Sorafenib is an oral multikinase inhibitor that targets growth signaling and angiogenesis in ccRCC by blocking VEGF-2-receptor (VEGFR-2), VEGF-3-receptor (VEGFR-3), PDGF-β-receptor (PDGFR-β), RAF- 1, c-Kit protein (c-Kit), and FMS-like tyrosine kinase 3 (Flt-3) (43). He et al. found that tumor-derived exosomes transferred drug resistance information from sorafenib-resistant ccRCC cells to non-resistant ccRCC cells by delivering miR-31-5p (44). At the same time, they further demonstrated that miR-31-5p promotes sorafenib resistance by targeting the 3’-UTR region of the MLH1 gene. But interestingly, Xuan et al. found that miR-549a expression was lower in TKI-resistant ccRCC cells and their derived exosomes compared with TKI-sensitive ccRCC, and they suggested that delivery of miR-549a to TKI-resistant renal cancer cells may reverse their own TKI resistance (31).

### 3.2 Exosomal lncRNA mediate ccRCC drug resistance

LncRNAs are noncoding RNAs greater than 200 nucleotides in length that have been found to be aberrantly expressed in various human cancer types and play important roles in tumorigenesis, metastasis, and drug resistance (45–47). Sunitinib, an oral multi-targeted TKI with a strong anti-angiogenic effect, is the first-line treatment for advanced ccRCC, but it is ineffective in a quarter of ccRCC patients, and most patients will relapse after 1 year of treatment (48, 49). Qu et al. found that an uncharacterized lncRNA was abundant in sunitinib-resistant ccRCC cell lines and their exosomes, and named it lncARSR. LncARSR can be secreted from resistant cells through exosomes to convert sunitinib-sensitive cells into resistant cells, thereby spreading drug resistance (50). Furthermore, they proved that lncARSR promotes sunitinib resistance by competitively binding to miR-34 and miR-449, leading to the upregulation of AXL/c-MET and activation of STAT3, AKT and ERK signaling pathways. In turn, activated AKT promotes lncARSR expression by inhibiting transcription factors FOXO1 and FOXO3a, forming a positive feedback loop. Greenberg et al. demonstrated that ketoconazole (KTZ) can reduce tumor-specific exosomes by inhibiting the protein expression of Alix, nSMase and Rab27a related to exosome biogenesis and secretion in ccRCC cells,reducing the delivery of substances from exosomes, which in turn enhances the efficacy of sunitinib and reduces the development of drug resistance (51).

### 3.3 Exosomal protein molecule mediate ccRCC drug resistance

It has been found that tumor-derived exosomes can mediate targeted drug resistance by delivering related protein molecules. Studies have shown that in breast cancer, exosomes derived from human breast cancer enamycin-resistant cells (MCF-7/ADM) are rich in P-gp and UCH-L1 proteins. MCF-7/ADM-derived exosomes can induce drug resistance in drug sensitive cells by internalizing and delivering P-gp and UCH-L1 to drug sensitive cells (52, 53). In ccRCC patients, Tsuruda et al. found that the protein expression level of RAB27B was significantly increased in sunitinib-resistant ccRCC cell lines. RAB27B is one of the main proteins involved in exosome secretion, and RAB27B may be involved in the formation of drug resistance through MAPK and VEGF signaling pathways (54). Wang et al. found that ccRCC cell-derived exosomes can help tumor cells evade immune killing and develop drug resistance through the mTOR-ERK-STAT-NF-κB protein signaling pathway (14).

## 4 Exosomes offer potential targets for early diagnosis of ccRCC

As the study of tumor-derived exosomes in ccRCC progresses, the value of tumor-derived exosomes as potential targets in the diagnosis of ccRCC is becoming increasingly evident. With the widespread application of imaging techniques, advances in surgical techniques, and improvements in pathological examinations, the early diagnosis of ccRCC has increased, and the prognosis of ccRCC has also improved (55). Although new or recurrent tumors can be diagnosed clinically by abdominal and chest CT, 30% of patients with ccRCC, when detected, already develop metastases (4–6). Once metastasis occurs, the cure rate of ccRCC will be greatly reduced, which is one of the important reasons hindering the further improvement of the cure rate of ccRCC. Therefore, there is a great need to have specific biomarkers suitable for ccRCC screening and monitoring as a supplement to imaging diagnosis. From a clinical standpoint, liquid biopsies are preferred over tissue biopsies because they are less invasive. Therefore, screening candidate biomarkers from body fluids such as serum or urine is crucial. A previous study reported that VEGF, VEGFR2, and CA9 regulated by HIF-1α were important biomarkers in liquid biopsies of ccRCC (56). In recent years, exosomes have become a new source of non-invasive tumor biomarkers. The bilayer membrane structure of the exosome is able to resist exogenous RNases and proteases, which produce more stable mRNAs, miRNAs, and intracellular functional proteins, making the exosome a sensitive marker for disease diagnosis (57). Additionally, exosomes derived from tumor cells are abundant in blood, urine and other body fluids, and have the advantages of easy access, non-invasive examination and tumor specificity (58). Studies have demonstrated the potential of exosomes as diagnostic markers for bladder cancer, pancreatic cancer, liver cancer and other tumors (59–61). In the serum and urine of ccRCC patients, the cargo of tumor-derived exosomes, for example, a series of miRNAs, can also be used as biological markers of ccRCC to provide meaningful targets for the early diagnosis and monitoring of ccRCC (**Table 1**).

**Table 1:** List of some original articles cited in this section, with the main results summarized.

Reference	Evaluationmethods	Number of patient samples	Results
**Zhang et al.** (15)	qRT-PCR	Serum of 82 ccRCC patients and 80 healthy controls	Exosomal miR-210 and exosomal miR-1233 significantly higher in each stage of ccRCC compared to controls
**Wang et al.** (62)	qRT-PCR	Serum of 45 ccRCC patients (including 5 patients with lung metastases) and 30 healthy controls	Exosomal miR-210 is upregulated in ccRCC, especially in patients with advanced tumor stage, high Fuhrman grade and metastases.
**Fujii et al.** (63)	qRT-PCR	Serum of 108 ccRCC patients	Exosomal miR-224 is highly expressed in ccRCC and is associated with poor prognosis.
**Horie et al.** (64).	Western Blot	Serum of 28 RCC patients	Serum exosomal GGT activity was significantly increased in patients with advanced RCC and in patients with microvascular invasion.
**Butz et al.** (65)	RT-PCR	Urine of 81 ccRCC patients, 24 patients with benign lesions, and 33 healthy controls	Combination of exosomal miR-126-3p and miR-449a in urine can discriminate healthy and ccRCC patients with high sensitivity.
**Song et al.** (66)	qRT-PCR	Urine of 70 early-stage (T1aN0M0) ccRCC patients and 30 healthy controls	The expression level of miR-30c-5p in urinary exosomes of ccRCC patients was significantly lower than that of controls, and the overexpression of miR-30c-5p inhibited ccRCC progression *in vitro* and *in vivo*.
**Zhao et al.** (67)	qRT-PCR/Western Blot	Gene expression samples were obtained from 533 ccRCC samples of The Cancer Genome Atlas (TCGA). Blood, urine and tumor tissue samples were collected from 4 ccRCC patients.	PTRF could be detected in the exosomes of the urine samples of ccRCC patients, which was significantly higher than that of the normal control group, and the expression of PTRF was significantly decreased after surgery. PTRF is regulated by the SHC1 gene through the EGFR pathway.

### 4.1 Exosomal biomarkers in serum

Exosomes in the serum of ccRCC patients can serve as a new source of ccRCC biomarkers. Zhang et al. found that ccRCC patients with different TNM stages had significantly higher expression levels of exosomal miR-210 and miR-1233 in their serum than healthy controls, and significantly lower expression levels of exosomal miR-210 and miR-1233 after surgery. They demonstrated that exosomal miR-210 and miR-1233 in serum may be used for liquid biopsy and contribute to the diagnosis and monitoring of ccRCC patients (15). Wang et al. also demonstrated that serum exosomal miR-210 was upregulated in ccRCC, especially in patients with advanced tumor stage, high Fuhrman grade, and metastases. Patients with ccRCC overexpressing miR-210 have a shorter chance of disease recurrence and survival time. Meanwhile, they found that exosomal miR-210 was superior to serum miR-210 in detecting ccRCC and was a good prognostic biomarker (62). Fujii et al. found that high levels of exosomal miR-224 in the serum of ccRCC patients were associated with poor patient prognosis (63). They demonstrated that exosomal miR-224 in the serum of ccRCC patients may be a promising prognostic biomarker for detecting microinvasion or tumor metastasis after surgery in ccRCC patients. In addition, studies have found that γ-glutamyltransferase (GGT) activity in serum exosomes is significantly elevated in patients with advanced RCC and distant metastases, as well as in patients with microvascular invasion (64). Exosomal GGT activity in serum may be a clinically useful marker for advanced ccRCC patients, and its combined use with conventional diagnostic modalities may improve the diagnosis of ccRCC patients. In a recent study, exosomal MYO15A was found to be significantly elevated in the serum of ccRCC patients, associated with a poorer prognosis, and may be a diagnostic target for ccRCC (68).

### 4.2 Exosomal biomarkers in urine

Exosomes in the urine of ccRCC patients can also serve as a new source of ccRCC biomarkers. A urine sample is preferable to a blood sample because it is anatomically close to the kidney and is one of the most readily available body fluids. Butz et al. found that the combination of exosomal miR-126-3p and miR-449a in urine can discriminate healthy people from ccRCC patients with high sensitivity. They demonstrated the potential utility of urinary exosomal miRNAs as a potential diagnostic tool for ccRCC, especially for small renal masses (65). Some studies have found that in ccRCC patients, urinalysis of exosomal miRNAs may be more suitable for selecting down-regulated miRNAs as biomarkers. Many of these down-regulated miRNAs, such as miR-205, may have large differences in expression levels between tumor and non-tumor cells, which helps make ccRCC easier to detect (69). Song et al. found that the expression level of miR-30c-5p in urinary exosomes of ccRCC patients was significantly lower than that in healthy controls. Furthermore, they discovered that miR-30c-5p could inhibit the progression of ccRCC by targeting heat shock protein 5 (HSPA5). Their study further demonstrated that urinary exosomal miR-30c-5p could be used as a highly specific and sensitive biomarker for the diagnosis and monitoring of ccRCC progression (66). Zhao et al. found that polymerase I and transcription release factor (PTRF) in urinary exosomes may also be potential biomarkers for ccRCC (67). PTRF could be detected in exosomes in urine samples of ccRCC patients, and was significantly higher than that in normal individuals. At the same time, the expression of PTRF was significantly decreased after operation. They demonstrated that PTRF is regulated by the SHC1 gene through the epidermal growth factor receptor (EGFR) pathway. By high-throughput sequencing, MB et al. found five novel mRNAs specific for stage I ccRCC in urinary exosomes, namely NME2, AAMP, CAPNS1, VAMP8, and MYL12B (70). In addition, a pilot study on the development of a reliable technique to detect exosomal CA9 in the urine of CCRCC patients for molecular diagnosis of CCRCC is underway in France (71).

## 5 Conclusion and prospect

Tumor-derived exosomes have received increasing attention in tumor research due to their role in intracellular communication during tumor progression. But compared with other cancer types, ccRCC has been relatively neglected in this research hotspot. In this review, we introduced the biological roles and research progress of tumor-derived exosomes in different aspects such as PMN formation, tumor angiogenesis, and EMT in the progression of ccRCC metastasis. Meanwhile, tumor-derived exosomes also play an important role in the development of ccRCC drug resistance. In ccRCC patients, tumor-derived exosomes can mediate drug resistance by delivering miRNA, lncRNA, and protein molecules. However, it is worth noting that the detailed mechanism of action of tumor-derived exosomes in ccRCC metastasis and drug resistance development remains to be further elucidated. In addition, the application of tumor-derived exosomes in ccRCC liquid biopsy holds great promise. Due to the encapsulation of the lipid bilayer membrane, the bioactive molecules within exosomes are not degraded by exogenous nucleases or proteases and are stable in biological fluids (72). A series of cargoes delivered by exosomes, especially a series of miRNAs, can be used as biomarkers of ccRCC, providing meaningful targets for early diagnosis and monitoring of ccRCC. Of course, the molecular characterization of each specific subtype of diagnostic markers is relevant and should be acknowledged like it has be done for urothelial malignancies (73). Meanwhile, miRNAs with high specificity and sensitivity in urinary exosomes need to be further discovered and identified, and simple biological tests for the diagnosis and monitoring of ccRCC need to be developed. At present, there are few studies on tumor-derived exosomes in the early monitoring and drug resistance development of ccRCC. In the future, more and more studies will focus on the application of tumor-derived exosomes in ccRCC liquid biopsy and treatment. In conclusion, a lot of work needs to be done to better understand the role of tumor-derived exosomes in ccRCC metastasis, drug resistance and diagnosis, and to make the potential clinical utility of tumor-derived exosomes in ccRCC a reality.

## Author contributions

All authors conceived and refined the idea. TJ conducted the literature searches and drafted the paper. ZZ, JZ refined the details of the article. MC and SC provided expert advice and support throughout. All authors contributed to the article and approved the submitted version.

## References

[1] Sung H Ferlay J Siegel RL Laversanne M Soerjomataram I Jemal A. Global cancer statistics 2020: GLOBOCAN estimates of incidence and mortality worldwide for 36 cancers in 185 countries. CA: A Cancer J Clin (2021) 71(3):209–49. doi: 10.3322/caac.21660 33538338

[2] Williamson SR Gill AJ Argani P Chen YB Egevad L Kristiansen G. Report from the international society of urological pathology (ISUP) consultation conference on molecular pathology of urogenital cancers: III: Molecular pathology of kidney cancer. Am J Surg Pathol (2020) 44(7):e47-e65. doi: 10.1097/PAS.0000000000001476 32251007 PMC7289677

[3] Chung BI Leow JJ Gelpi-Hammerschmidt F Wang Y Del Giudice F De S. Racial disparities in postoperative complications after radical nephrectomy: A population-based analysis. Urol Jun (2015) 85(6):1411–6. doi: 10.1016/j.urology.2015.03.001 25881864

[4] Barata PC Rini BI. Treatment of renal cell carcinoma: Current status and future directions. Ca-a Cancer J Clin (2017) 67(6):507–24. doi: 10.3322/caac.21411 28961310

[5] Marona P Gorka J Mazurek Z Wilk W Rys J Majka M. MCPIP1 downregulation in clear cell renal cell carcinoma promotes vascularization and metastatic progression. Cancer Res (2017) 77(18):4905–20. doi: 10.1158/0008-5472.Can-16-3190 28716897

[6] Shingarev R Jaimes EA. Renal cell carcinoma: new insights and challenges for a clinician scientist. Am J Physiology-Renal Physiol (2017) 313(2):F145–54. doi: 10.1152/ajprenal.00480.2016 PMC5582896 28381462

[7] Pal K Madamsetty VS Dutta SK Wang E Angom RS Mukhopadhyay D. Synchronous inhibition of mTOR and VEGF/NRP1 axis impedes tumor growth and metastasis in renal cancer. NPJ Precis Oncol (2019) 3:31. doi: 10.1038/s41698-019-0105-2 31840081 PMC6895165

[8] Escudier B Porta C Schmidinger M Rioux-Leclercq N Bex A Khoo V. Renal cell carcinoma: ESMO clinical practice guidelines for diagnosis, treatment and follow-up. Ann Oncol May (2019) 30(5):706–20. doi: 10.1093/annonc/mdz056 30788497

[9] Himbert D Zeuschner P Ayoubian H Heinzelmann J Stockle M Junker K. Characterization of CD147, CA9, and CD70 as tumor-specific markers on extracellular vesicles in clear cell renal cell carcinoma. Diagnostics (2020) 10(12):1034. doi: 10.3390/diagnostics10121034 33276608 PMC7761541

[10] Colombo M Raposo G Thery C. Biogenesis, secretion, and intercellular interactions of exosomes and other extracellular vesicles. Annu Rev Cell Dev Biol (2014) 30:255–89. doi: 10.1146/annurev-cellbio-101512-122326 25288114

[11] Kalluri R LeBleu VS. The biology, function, and biomedical applications of exosomes. Science (2020) 367(6478):640–+. eaau6977. doi: 10.1126/science.aau6977 PMC7717626 32029601

[12] Fu W Zhao H Liu Y Nie H Gao B Yin F. Exosomes derived from cancer-associated fibroblasts regulate cell progression in clear-cell renal-cell carcinoma. Nephron (2021) 146(4):383–92. doi: 10.1159/000520304 34903693

[13] Wang L Yang G Zhao D Wang J Bai Y Peng Q. CD103-positive CSC exosome promotes EMT of clear cell renal cell carcinoma: role of remote MiR-19b-3p (vol 18, 86, 2019). Mol Cancer (2020) 19(1):144. doi: 10.1186/s12943-020-01261-y 32972401 PMC7513539

[14] Wang X Shi Q Cui L Wang K Gong P He X. Tumor-derived exosomes facilitate tumor cells escape from drug therapy in clear cell renal cell carcinoma. Trans Cancer Res (2020) 9(5):3416–25. doi: 10.21037/tcr-19-2246 PMC8798292 35117707

[15] Zhang W Ni M Su Y Wang H Zhu S Zhao A. MicroRNAs in serum exosomes as potential biomarkers in clear-cell renal cell carcinoma. Eur Urol Focus (2018) 4(3):412–9. doi: 10.1016/j.euf.2016.09.007 28753793

[16] Zhang L Wu X Luo C Chen X Yang L Tao J. The 786-0 renal cancer cell-derived exosomes promote angiogenesis by downregulating the expression of hepatocyte cell adhesion molecule. Mol Med Rep (2013) 8(1):272–6. doi: 10.3892/mmr.2013.1458 23652371

[17] Ribatti D Mangialardi G Vacca A. Stephen Paget and the ‘seed and soil’ theory of metastatic dissemination. Clin Exp Med (2006) 6(4):145–9. doi: 10.1007/s10238-006-0117-4 17191105

[18] Peinado H Zhang H Matei IR Costa-Silva B Hoshino A Rodrigues G. Pre-metastatic niches: organ-specific homes for metastases. Nat Rev Cancer May (2017) 17(5):302–17. doi: 10.1038/nrc.2017.6 28303905

[19] Zeng Z Li Y Pan Y Lan X Song F Sun J. Cancer-derived exosomal miR-25-3p promotes pre-metastatic niche formation by inducing vascular permeability and angiogenesis. Nat Commun (2018) 9(1):5395. doi: 10.1038/s41467-018-07810-w 30568162 PMC6300604

[20] Grange C Tapparo M Collino F Vitillo L Damasco C Deregibus MC. Microvesicles released from human renal cancer stem cells stimulate angiogenesis and formation of lung premetastatic niche. Cancer Res (2011) 71(15):5346–56. doi: 10.1158/0008-5472.Can-11-0241 21670082

[21] You Y Ren Y Liu J Qu J. Promising epigenetic biomarkers associated with cancer-Associated-Fibroblasts for progression of kidney renal clear cell carcinoma. Front Genet (2021) 12:736156. doi: 10.3389/fgene.2021.736156 34630525 PMC8495159

[22] Liu Y Fu W Cao X Li S Xiong T Zhang X. Delivery of miR-224-5p by exosomes from cancer-associated fibroblasts potentiates progression of clear cell renal cell carcinoma. Comput Math Methods Med (2021) 2021:5517747. doi: 10.1155/2021/5517747 34122615 PMC8169240

[23] Ding M Zhao X Chen X Diao W Kan Y Cao W. Cancer-associated fibroblasts promote the stemness and progression of renal cell carcinoma *via* exosomal miR-181d-5p. Cell Death Discovery (2022) 8(1):439. doi: 10.1038/s41420-022-01219-7 36319622 PMC9626570

[24] Huang X Wang J Guan J Zheng Z Hao J Sheng Z. Exosomal Circsafb2 reshaping tumor environment to promote renal cell carcinoma progression by mediating M2 macrophage polarization. Front Oncol (2022) 12:808888. doi: 10.3389/fonc.2022.808888 35646637 PMC9133324

[25] Yang H Zhang H Ge S Ning T Bai M Li J. Exosome-derived miR-130a activates angiogenesis in gastric cancer by targeting c-MYB in vascular endothelial cells. Mol Ther (2018) 26(10):2466–75. doi: 10.1016/j.ymthe.2018.07.023 PMC6171076 30120059

[26] Huang X-Y Huang Z-L Huang J Xu B Huang XY Xu YH. Exosomal circRNA-100338 promotes hepatocellular carcinoma metastasis via enhancing invasiveness and angiogenesis. J Exp Clin Cancer Res (2020) 39(1):20. doi: 10.1186/s13046-020-1529-9 31973767 PMC6979009

[27] Shang D Xie C Hu J Tan J Yuan Y Liu Z. Pancreatic cancer cell-derived exosomal microRNA-27a promotes angiogenesis of human microvascular endothelial cells in pancreatic cancer *via* BTG2. J Cell Mol Med (2020) 24(1):588–604. doi: 10.1111/jcmm.14766 31724333 PMC6933412

[28] Hou Y Fan L Li H. Oncogenic miR-27a delivered by exosomes binds to SFRP1 and promotes angiogenesis in renal clear cell carcinoma. Mol Therapy-Nucleic Acids (2021) 24:92–103. doi: 10.1016/j.omtn.2020.11.019 PMC7941030 33738141

[29] Li Y-L Wu L-W Zeng L-H Zhang ZY Wang W Zhang C. ApoC1 promotes the metastasis of clear cell renal cell carcinoma *via* activation of STAT3. Oncogene (2020) 39(39):6203–17. doi: 10.1038/s41388-020-01428-3 32826950

[30] Horie K Kawakami K Fujita Y Sugaya M Kameyama K Mizutani K. Exosomes expressing carbonic anhydrase 9 promote angiogenesis. Biochem Biophys Res Commun (2017) 492(3):356–61. doi: 10.1016/j.bbrc.2017.08.107 28851650

[31] Xuan Z Chen C Tang W Ye S Zheng J Zhao Y. TKI-resistant renal cancer secretes low-level exosomal miR-549a to induce vascular permeability and angiogenesis to promote tumor metastasis. Front Cell Dev Biol (2021) 9:689947. doi: 10.3389/fcell.2021.726535 34179017 PMC8222687

[32] Zhang W Zheng X Yu Y Zheng L Lan J Wu Y. Renal cell carcinoma-derived exosomes deliver lncARSR to induce macrophage polarization and promote tumor progression *via* STAT3 pathway. Int J Biol Sci (2022) 18(8):3209–22. doi: 10.7150/ijbs.70289 PMC9134902 35637970

[33] Mittal V. Epithelial mesenchymal transition in tumor metastasis. Annual review of pathology (2018) 13:395–412. doi: 10.1146/annurev-pathol-020117-043854 29414248

[34] He S Li Z Yu Y Zeng Q Cheng Y Ji W. Exosomal miR-499a-5p promotes cell proliferation, migration and EMT *via* mTOR signaling pathway in lung adenocarcinoma. Exp Cell Res Jun 15 (2019) 379(2):203–13. doi: 10.1016/j.yexcr.2019.03.035 30978341

[35] You X Wang Y Meng J Han S Liu L Sun Y. Exosomal miR663b exposed to TGFbeta1 promotes cervical cancer metastasis and epithelialmesenchymal transition by targeting MGAT3. Oncol Rep (2021) 45(4):12. doi: 10.3892/or.2021.7963 33649791 PMC7877003

[36] Li D-Y Lin F-F Li G-P Zeng F-C. Exosomal microRNA-15a from ACHN cells aggravates clear cell renal cell carcinoma *via* the BTG2/PI3K/AKT axis. Kaohsiung J Med Sci Nov (2021) 37(11):973–82. doi: 10.1002/kjm2.12428 PMC11896453 34337864

[37] Hu G Ma J Zhang J Chen Y Liu H Huang Y. Hypoxia-induced lncHILAR promotes renal cancer metastasis *via* ceRNA for the miR-613/206/1-1-3p/Jagged-1/Notch/CXCR4 signaling pathway. Mol Ther (2021) 29(10):2979–94. doi: 10.1016/j.ymthe.2021.05.020 PMC8531137 34058384

[38] Tenold M Ravi P Kumar M Bowman A Hammers H Choueiri TK. Current approaches to the treatment of advanced or metastatic renal cell carcinoma. Am Soc Clin Oncol Educ book Am Soc Clin Oncol Annu Meeting 2020-Mar (2020) 40:1–10. doi: 10.1200/edbk_279881 32239988

[39] Wyler L Napoli CU Ingold B Sulser T Heikenwälder M Schraml P. Brain metastasis in renal cancer patients: metastatic pattern, tumour-associated macrophages and chemokine/chemoreceptor expression. Br J Cancer Feb 4 (2014) 110(3):686–94. doi: 10.1038/bjc.2013.755 PMC3915122 24327013

[40] Sun W J-d L Jiang H Duan DD. Tumor exosomes: a double-edged sword in cancer therapy. Acta Pharmacolo Sin Apr (2018) 39(4):534–41. doi: 10.1038/aps.2018.17 PMC5888693 29542685

[41] Wang J Zeng H Zhang H Han Y. The role of exosomal PD-L1 in tumor immunotherapy. Trans Oncol (2021) 14(5):101047. doi: 10.1016/j.tranon.2021.101047 PMC7921878 33647542

[42] Garje R An J Greco A Vaddepally RK Zakharia Y. The future of immunotherapy-based combination therapy in metastatic renal cell carcinoma. Cancers (2020) 12(1):143. doi: 10.3390/cancers12010143 31936065 PMC7017064

[43] Yu X Guo G Li X Zhang C Huang L Fang D. Retrospective analysis of the efficacy and safety of sorafenib in Chinese patients with metastatic renal cell carcinoma and prognostic factors related to overall survival. Medicine (2015) 94(34):e1361. doi: 10.1097/md.0000000000001361 26313773 PMC4602909

[44] He J He J Min L He Y Guan H Wang J. Extracellular vesicles transmitted miR-31-5p promotes sorafenib resistance by targeting MLH1 in renal cell carcinoma. Int J Cancer (2020) 146(4):1052–63. doi: 10.1002/ijc.32543 31259424

[45] Meseure D Alsibai KD Nicolas A Bieche I Morillon A. Long noncoding RNAs as new architects in cancer epigenetics, prognostic biomarkers, and potential therapeutic targets. BioMed Res Int (2015) 2015:2015320214. doi: 10.1155/2015/320214 PMC4584070 26448935

[46] Lv H Lv G Han Q Yang W Wang H. Noncoding RNAs in liver cancer stem cells: The big impact of little things. Cancer Lett (2018) 418:51–63. doi: 10.1016/j.canlet.2018.01.001 29307614

[47] Yan H Bu P. Non-coding RNAs in cancer stem cells. Cancer Lett (2018) 2018:421:121–126. doi: 10.1016/j.canlet.2018.01.027 29331418

[48] Duran I Lambea J Maroto P González-Larriba JL Flores L Granados-Principal S. Resistance to targeted therapies in renal cancer: The importance of changing the mechanism of action. Targeted Oncol Feb (2017) 12(1):19–35. doi: 10.1007/s11523-016-0463-4 27844272

[49] Bridgeman VL Wan E Foo S Nathan MR Welti JC Frentzas S. Preclinical evidence that trametinib enhances the response to antiangiogenic tyrosine kinase inhibitors in renal cell carcinoma. Mol Cancer Ther (2016) 15(1):172–83. doi: 10.1158/1535-7163.Mct-15-0170 26487278

[50] Qu L Ding J Chen C Wu ZJ Liu B Gao Y. Exosome-transmitted lncARSR promotes sunitinib resistance in renal cancer by acting as a competing endogenous RNA. Cancer Cell (2016) 29(5):653–68. doi: 10.1016/j.ccell.2016.03.004 27117758

[51] Greenberg JW Kim H Moustafa AA Datta A Barata PC Boulares AH. Repurposing ketoconazole as an exosome directed adjunct to sunitinib in treating renal cell carcinoma. Sci Rep (2021) 11(1):10200. doi: 10.1038/s41598-021-89655-w 33986386 PMC8119955

[52] Lv M-M Zhu X-Y Chen W-X Zhong SL Hu Q Ma TF. Exosomes mediate drug resistance transfer in MCF-7 breast cancer cells and a probable mechanism is delivery of p-glycoprotein. Tumor Biol (2014) 35(11):10773–9. doi: 10.1007/s13277-014-2377-z 25077924

[53] Ning K Wang T Sun X Zhang P Chen Y Jin J. UCH-L1-containing exosomes mediate chemotherapeutic resistance transfer in breast cancer. J Surg Oncol (2017) 115(8):932–40. doi: 10.1002/jso.24614 28334432

[54] Tsuruda M Yoshino H Okamura S Kuroshima K Osako Y Sakaguchi T. Oncogenic effects of RAB27B through exosome independent function in renal cell carcinoma including sunitinib-resistant. PloS One (2020) 15(5):e0232545. doi: 10.1371/journal.pone.0232545 32379831 PMC7205224

[55] Greef B Eisen T. Medical treatment of renal cancer: new horizons. Br J Cancer (2016) 115(5):505–16. doi: 10.1038/bjc.2016.230 PMC4997553 27490806

[56] Spirina LV Usynin YA Yurmazov ZA Slonimskaya EM Kolegova ES Kondakova IV. Transcription factors NF-kB, HIF-1, HIF-2, growth factor VEGF, VEGFR2 and carboanhydrase IX mRNA and protein level in the development of kidney cancer metastasis. Mol Biol (2017) 51(2):328–32. doi: 10.1134/s0026893317020194 28537244

[57] Xu L Gimple RC Lau WB Lau B Fei F Shen Q. The present and future of the mass spectrometry-based investigation of the exosome landscape. Mass Spectromet Rev (2020) 39(5-6):745–62. doi: 10.1002/mas.21635 32469100

[58] Mao W Wang K Wu Z Xu B Chen M. Current status of research on exosomes in general, and for the diagnosis and treatment of kidney cancer in particular. J Exp Clin Cancer Res (2021) 40(1):305. doi: 10.1186/s13046-021-02114-2 34583759 PMC8477471

[59] Elsharkawi F Elsabah M Shabayek M Khaled H. Urine and serum exosomes as novel biomarkers in detection of bladder cancer. Asian Pac J Cancer Prevention: APJCP (2019) 20(7):2219–24. doi: 10.31557/apjcp.2019.20.7.2219 PMC6745236 31350988

[60] Chen J Yao D Chen W Li Z Guo Y Zhu F. Serum exosomal miR-451a acts as a candidate marker for pancreatic cancer. Int J Biol Markers (2022) 37(1):74–80. doi: 10.1177/17246008211070018 35001683

[61] Wei X-C Liu L-J Zhu F. Exosomes as potential diagnosis and treatment for liver cancer. World J Gastrointest Oncol (2022) 14(1):334–47. doi: 10.4251/wjgo.v14.i1.334 PMC8790408 35116120

[62] Wang X Wang T Chen C Wu Z Bai P Li S. Serum exosomal miR-210 as a potential biomarker for clear cell renal cell carcinoma. J Cell Biochem (2019) 120(2):1492–502. doi: 10.1002/jcb.27347 30304555

[63] Fujii N Hirata H Ueno K Mori J Oka S Shimizu K. Extracellular miR-224 as a prognostic marker for clear cell renal cell carcinoma. Oncotarget (2017) 8(66):109877–88. doi: 10.18632/oncotarget.22436 PMC5746350 29299115

[64] Horie K Kawakami K Fujita Y Matsuda Y Arai T Suzui N. Serum exosomal gamma-glutamyltransferase activity increased in patients with renal cell carcinoma with advanced clinicopathological features. Oncology (2020) 98(10):734–42. doi: 10.1159/000508688 32726790

[65] Butz H Nofech-Mozes R Ding Q Khella HWZ Szabó PM Jewett M. Exosomal MicroRNAs are diagnostic biomarkers and can mediate cell-cell communication in renal cell carcinoma. Eur Urol Focus (2016) 2(2):210–8. doi: 10.1016/j.euf.2015.11.006 28723537

[66] Song S Long M Yu G Cheng Y Yang Q Liu J. Urinary exosome miR-30c-5p as a biomarker of clear cell renal cell carcinoma that inhibits progression by targeting HSPA5. J Cell Mol Med (2019) 23(10):6755–65. doi: 10.1111/jcmm.14553 PMC6787446 31342628

[67] Zhao Y Wang Y Zhao E Tan Y Geng B Kang C. PTRF/CAVIN1, regulated by SHC1 through the EGFR pathway, is found in urine exosomes as a potential biomarker of ccRCC. Carcinogenesis (2020) 41(3):274–83. doi: 10.1093/carcin/bgz147 31605605

[68] Yoshino H Tatarano S Tamai M Tsuruda M Iizasa S Arima J. Exosomal microRNA-1 and MYO15A as a target for therapy and diagnosis in renal cell carcinoma. Biochem Biophys Res Commun (2022) 630:71–6. doi: 10.1016/j.bbrc.2022.09.056 36150242

[69] Crentsil VC Liu H Sellitti DF. Comparison of exosomal microRNAs secreted by 786-O clear cell renal carcinoma cells and HK-2 proximal tubule-derived cells in culture identifies microRNA-205 as a potential biomarker of clear cell renal carcinoma. Oncol Lett (2018) 16(1):1285–90. doi: 10.3892/ol.2018.8751 PMC6063036 30061948

[70] Marek-Bukowiec K Konieczny A Ratajczyk K Czapor-Irzabek H Gorniak A Kowal P. mRNA fingerprint of early-stage clear cell renal cell carcinoma identified in urinary exosomes by mRNA sequencing. Polish Arch Internal Medicine-Polskie Archiwum Medycyny Wewnetrznej (2021) 131(6):582–5. doi: 10.20452/pamw.16005 34018710

[71] Li G Mallouk N Flandrin P Garcin A Lambert C Berremila SA. Presence of urinary exosomes for liquid biopsy of clear cell renal cell carcinoma: Protocol for a pilot feasibility study. JMIR Res Protoc (2021) 10(7):e24423. doi: 10.2196/24423 34283029 PMC8335600

[72] Liu J Ren L Li S Li W Zheng X Yang Y. The biology, function, and applications of exosomes in cancer. Acta Pharm Sin B Sep (2021) 11(9):2783–97. doi: 10.1016/j.apsb.2021.01.001 PMC8463268 34589397

[73] Nicolazzo C Busetto GM Del Giudice F Sperduti I Giannarelli D Gradilone A. The long-term prognostic value of survivin expressing circulating tumor cells in patients with high-risk non-muscle invasive bladder cancer (NMIBC). J Cancer Res Clin Oncol (2017) 143(10):1971–6. doi: 10.1007/s00432-017-2449-8 PMC11819121 28555356

